# Supplementary material for: Combined Rho-kinase inhibition and immunogenic cell death triggers and propagates immunity against cancer
Source: Nat Commun. 2018 Jun 4;9:2165. doi: 10.1038/s41467-018-04607-9 (PMC5986820; doi:10.1038/s41467-018-04607-9)
Supplement: Supplementary file 1 — Supplementary Information [file 41467_2018_4607_MOESM1_ESM.pdf]

Supplementary Information

Combined Rho-kinase inhibition and immunogenic cell death triggers and propagates immunity against cancer

Gi-Hoon Nam, Eun-Jung Lee, Yoon Kyoung Kim, Yeonsun Hong, Yoonjeong Choi, Myung-Jeom Ryu, Jiwan Woo, Yakdol Cho, Dong June Ahn, Yoosoo Yang, Ick-Chan Kwon, Seung-Yoon Park, and In-San Kim

Contents

1. Supplementary Figure 1-21

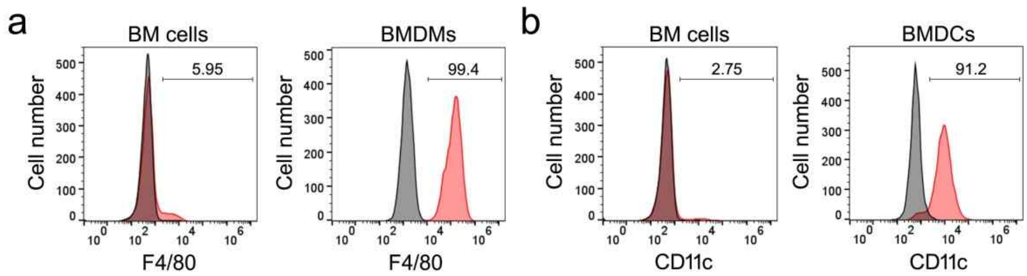

Supplementary Figure 1. Characterization of BMDMs and BMDCs. Bone marrow (BM) cells were isolated from 8-week-old male mice and differentiated into macrophages (BMDMs, **a**) or dendritic cells (BMDCs, **b**) by incubating with M-CSF or Flt3 ligand, respectively. Purity of BMDMs and BMDCs was verified by flow cytometry for F4/80 and CD11c expression, respectively. Representative results are shown. The gray peaks represent the isotype control.

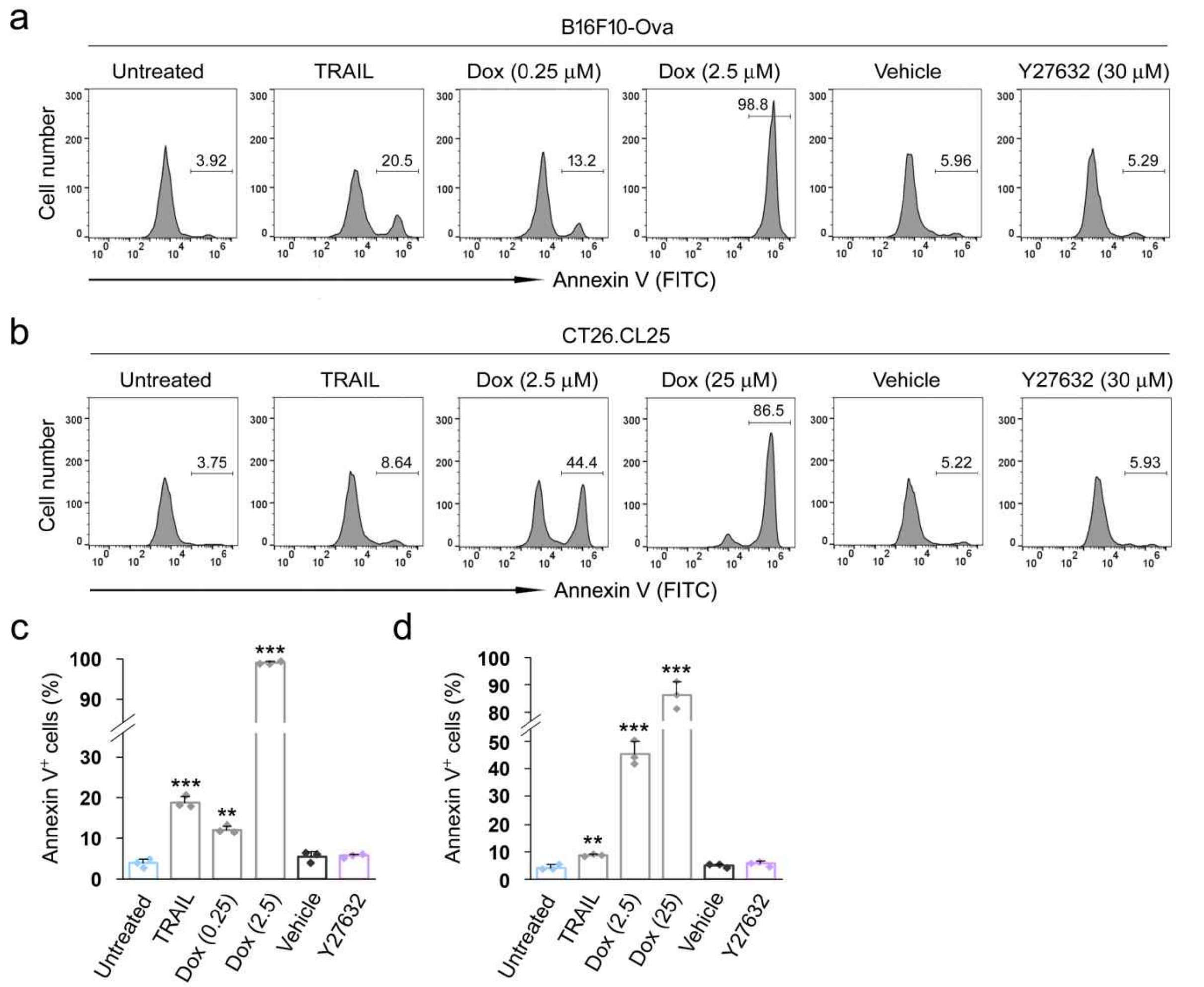

Supplementary Figure 2. Effect of ROCK blockade on cancer cell apoptosis. (**a**,**b**) B16F10-Ova (**a**,**c**) and CT26.CL25 (**b**,**d**) cells were incubated for 24 hours in the presence of Y27632 (30 μM), stained with Annexin-V-488, and analysed by flow cytometry. Recombinant mouse TRAIL (10 μg ml^{-1}) and doxorubicin were used as positive controls. (**a**,**b**) Representative results are shown (left panels). (**c**,**d**) The data are presented as the mean ± s.d. (n=3). $^{**}P < 0.01$, $^{***}P < 0.001$ compared with untreated group; significance determined by unpaired Student's *t*-test.

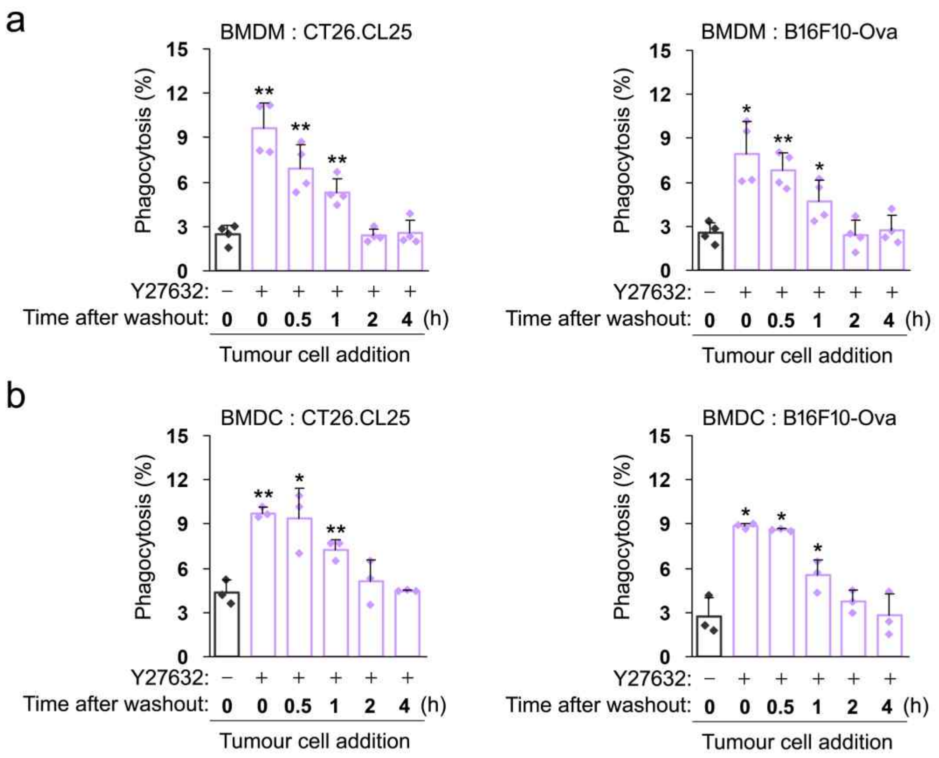

Supplementary Figure 3. The phagocytosis-promoting effect of Y27632 is reversible. CellTracker Deep Red-labeled BMDMs (**a**) or BMDCs (**b**) were pretreated with Y27632 (30 μM) for 1 hour. At 0, 0.5, 1, 2, or 4 hours after Y27632 washout, CMFDA-stained CT26.CL25 or B16F10-Ova cells were added into BMDMs or BMDCs and co-cultured for 2 hours, and phagocytosis (%) was analysed by flow cytometry. Data are presented as means ± s.d. (n=3–4). **P* < 0.05, ***P* < 0.01 compared with untreated group; significance determined by unpaired Student's *t*-test.

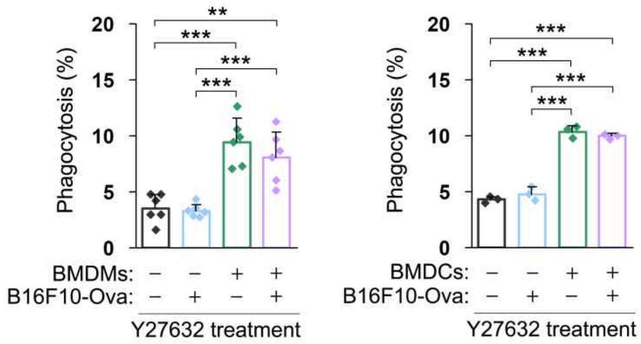

Supplementary Figure 4. ROCK blockade modulates the phagocytic ability of BMDMs and BMDCs. Phagocytes (BMDMs or BMDCs) and/or B16F10-Ova cells were pretreated with Y27632 (30 μM). Then, BMDMs or BMDCs were co-cultured with B16F10-Ova cells for 2 hours, and phagocytosis (%) was analysed by flow cytometry. Data are presented as means ± s.d. (n=3–6). $^{**}P < 0.01$, $^{***}P < 0.001$; significance determined by one-way ANOVA with Tukey's post-hoc test.

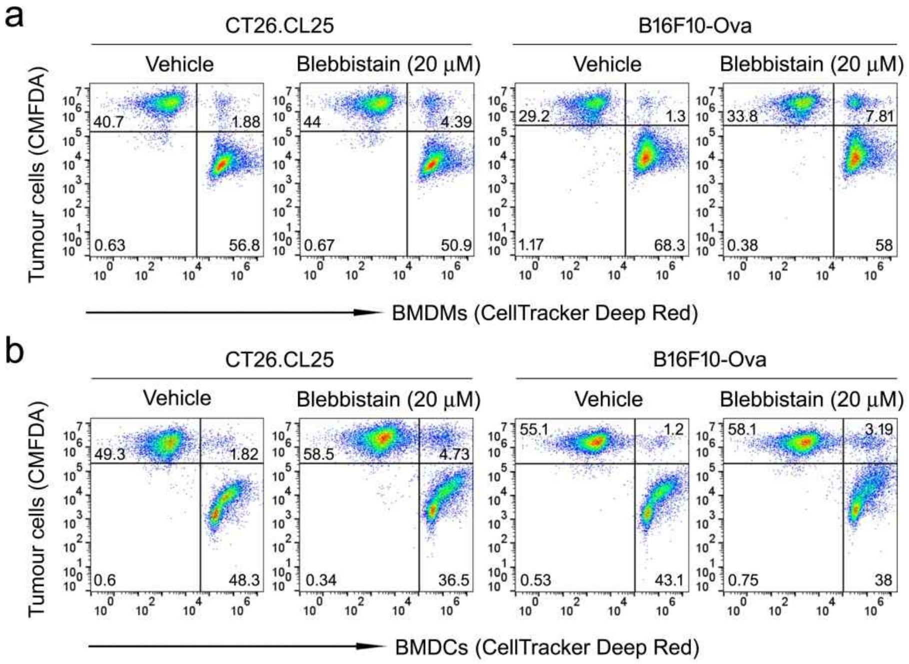

Supplementary Figure 5. Myosin II inhibitor enhances cancer cell phagocytosis. CellTracker Deep Red-labeled BMDMs (**a**) or BMDCs (**b**) were co-cultured with CMFDA-stained CT26.CL25 or B16F10-Ova cells for 2 hours in the presence of blebbistatin (20 μM) or vehicle, and phagocytosis (%) were analysed by flow cytometry. Upper right quadrants indicate BMDMs or BMDCs harboring cancer cells. Representative flow cytometry plots are shown.

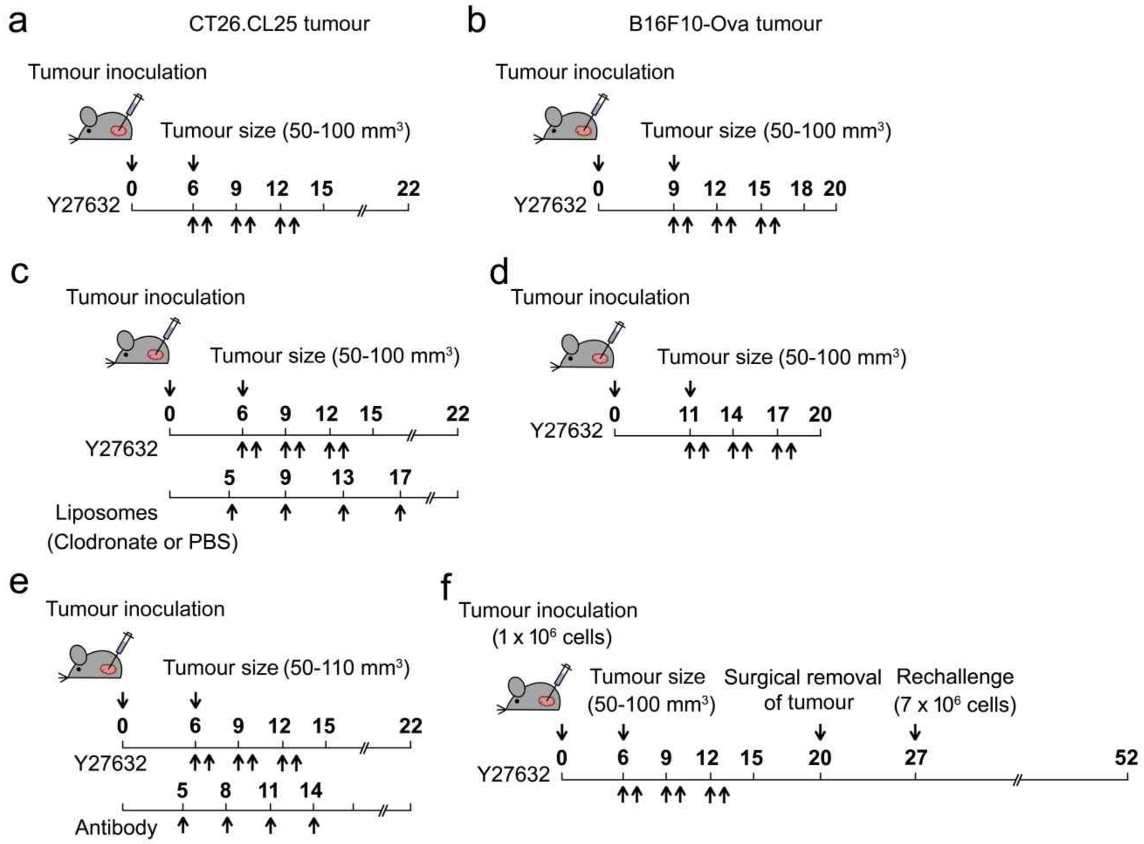

Supplementary Figure 6. Experimental schedules on tumour models. (**a**,**b**) Schematic diagram of experiments for investigating the effect of Y27632 on tumour growth in two syngeneic tumour models: CT26.CL25 tumour (**a**) and B16F10-Ova tumour (**b**). (**c**) Schematic diagram of experiments for investigating the effect of phagocyte depletion on antitumour activity of Y27632. (**d**) Schematic diagram of experiments for investigating the effect of Y27632 on tumour growth in CT26.CL25 tumour-bearing nude mice. (**e**) Schematic diagram of experiments for investigating the effect of Y27632 on tumour growth in the CT26.CL25 tumour model, where $CD4^+$ or $CD8^+$ T cells were depleted. (**f**) Schematic diagram of experiments for investigating the effect of Y27632 on antitumour immunity in CT26.CL25 tumour-bearing mice.

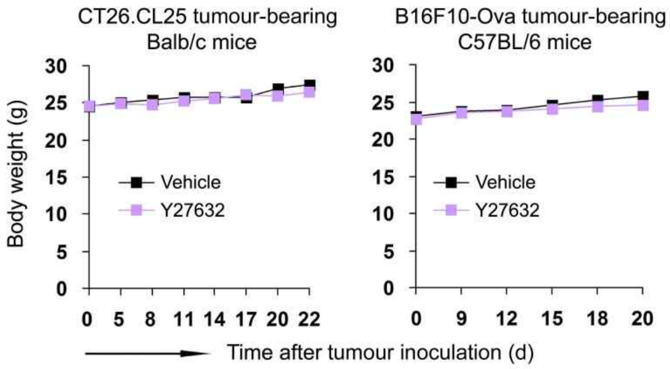

Supplementary Figure 7. Effect of ROCK blockade on body weight in tumour-bearing mice. CT26.CL25 tumour-bearing BALB/c mice (left panel) and B16F10-Ova tumour-bearing C57BL/6 mice (right panel) were injected (i.v.) with Y27632 (10 mg kg^{-1}) or vehicle (PBS) as indicated in Supplementary Fig. 6a and b. Body weight was measured at the indicated times. Data are presented as means ± s.e.m (CT26.CL25 model, n=17; B16F10-Ova model, n=17–19).

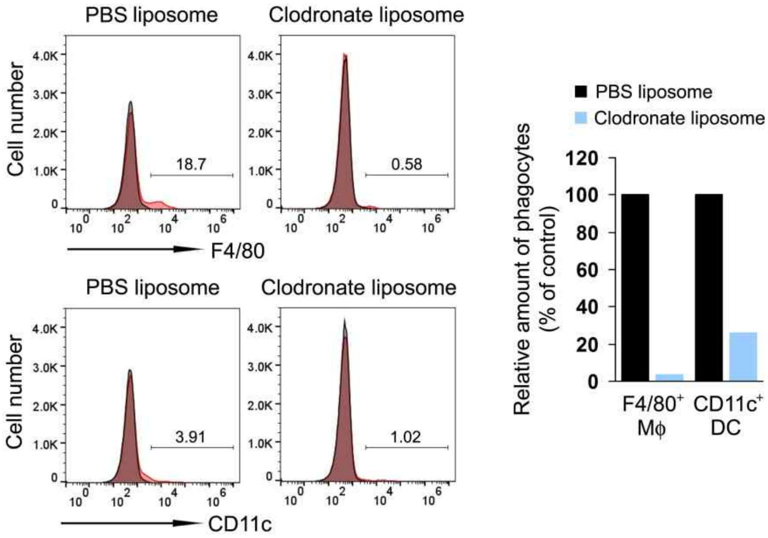

Supplementary Figure 8. Verification of phagocyte depletion in a mouse model. CT26.CL25-mCherry tumour-bearing mice were injected (i.v.) with Y27632 (10 mg kg^{-1}) together with clodronate liposomes or PBS liposomes. Splenocytes were isolated from each group of mice, and the percentages of F4/80^+ macrophages and CD11c^+ DCs were analysed by flow cytometry. A representative result is shown (left panels). The gray peaks represent the isotype control. Relative amounts of F4/80^+ macrophages and CD11c^+ DCs were calculated from left panels (right panel).

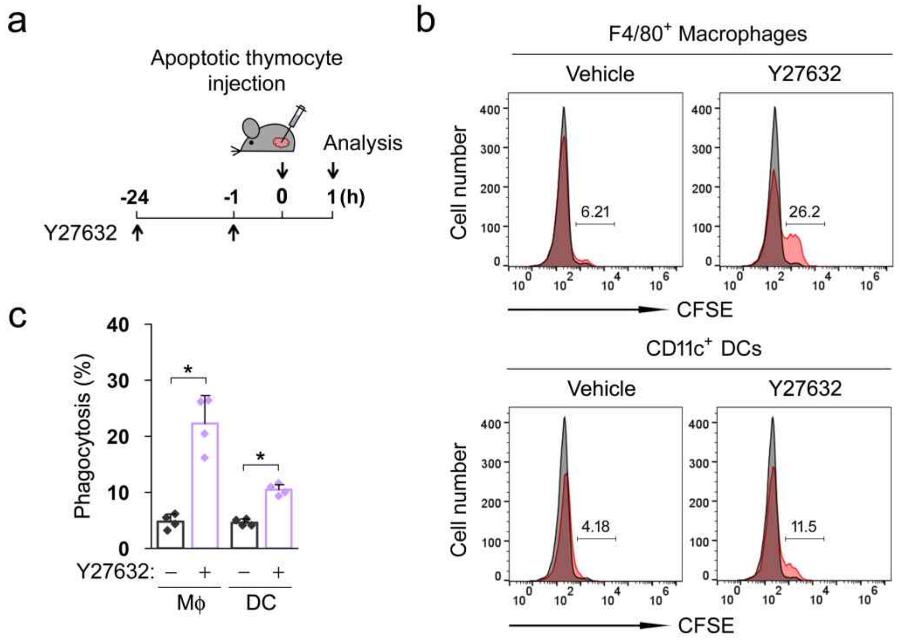

Supplementary Figure 9. Effect of ROCK blockade on apoptotic cell phagocytosis in normal mice. CFSE-stained apoptotic thymocytes (1×10^7) were injected (i.v.) into the 8-week-old C57BL/6 mice treated with Y27632 (10 mg kg^{-1}) as indicated in (**a**). After 1 hour, $F4/80^+$ macrophages and $CD11c^+$ DCs were isolated among splenocytes from the mice, and phagocytosis (%) were analysed by flow cytometry. The gray peaks represent CFSE levels in macrophages and DCs from mice without thymocyte injection. (**b**) Representative flow cytometry plots. (**c**) The percentage of phagocytosis. Data are presented as means ± s.d. (n=4). *$P < 0.05$; significance was determined by significance determined by Mann-Whitney test.

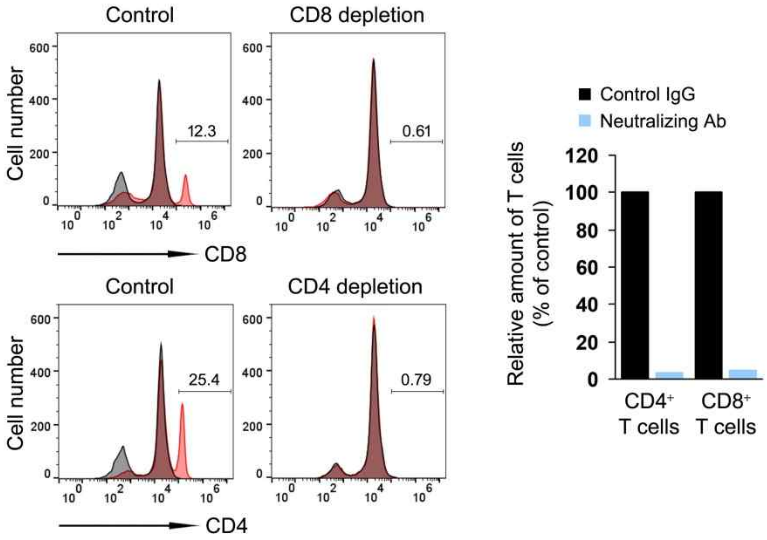

Supplementary Figure 10. Verification of T-cell depletion on a mouse model. CT26.CL25 tumour-bearing mice were injected (i.v.) with Y27632 (10 mg kg^{-1}) in conjunction with neutralizing anti-CD4 (clone GK1.5) or anti-CD8 antibody (clone 2.43). Rat IgG2b (clone LTF-2) was used as an isotype-matched control. Splenocytes were isolated from each group of mice, and the percentages of $CD4^{+}$ and $CD8^{+}$ T cells were analysed. A representative result is shown (left panels). The gray peaks represent the isotype control. Relative amounts of $CD4^{+}$ and $CD8^{+}$ T cells were calculated from left panels (right panel).

.

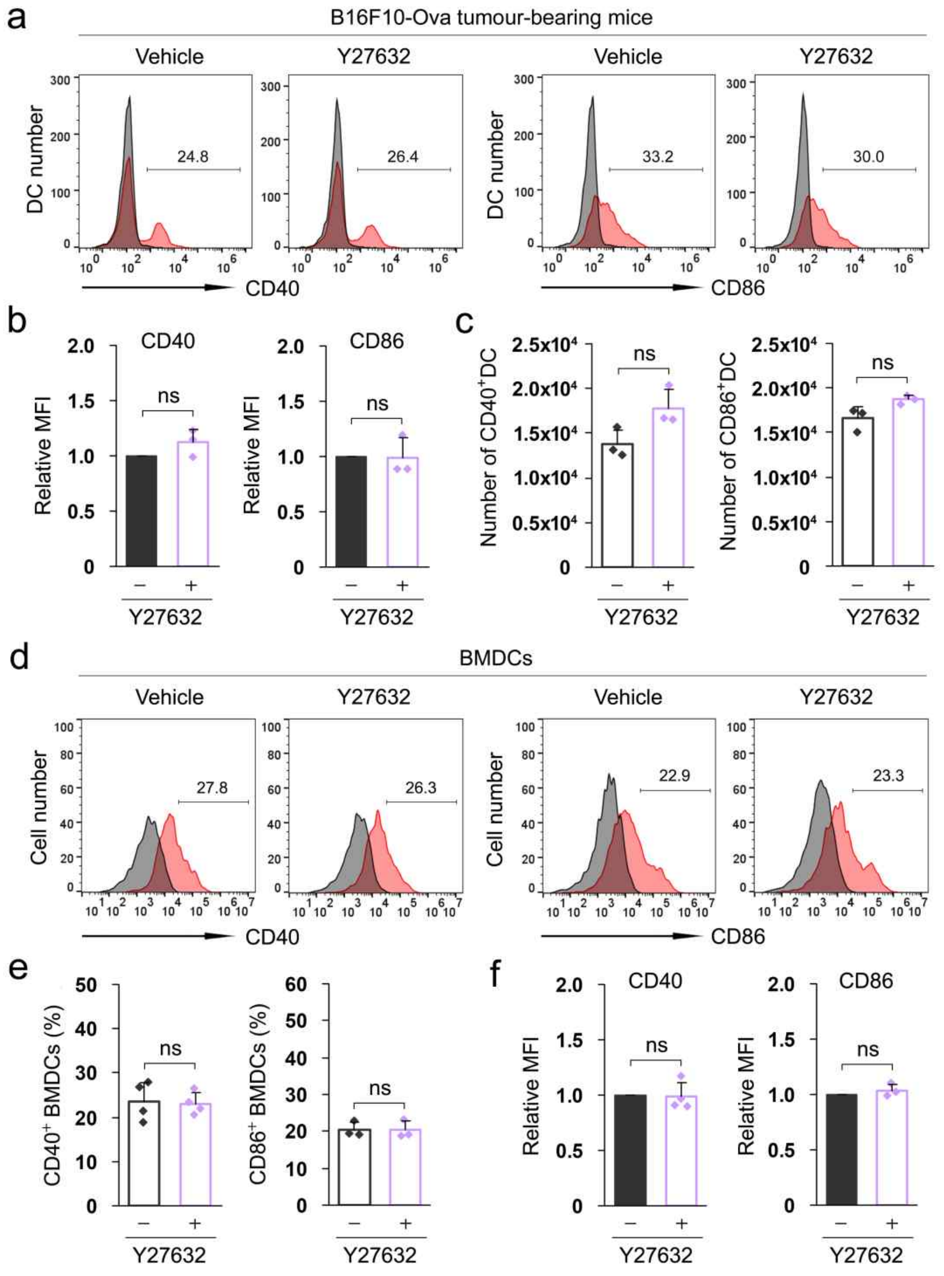

Supplementary Figure 11. ROCK blockade has no effect on DC maturation. (**a-c**) B16F10-Ova tumour-bearing mice were injected (i.v.) with Y27632 (10 mg kg^{-1}) or vehicle (PBS). Twenty days after tumour inoculation, cells from tumour draining LNs were isolated, and the percentage of $CD40^+$ or $CD86^+$ DCs was analysed by flow cytometry. (**a**) Representative results are shown. The gray peaks represent the isotype control. (**b**) Relative mean fluorescence intensity (MFI) and (**c**) absolute cell number of $CD40^+$ or $CD86^+$ DCs are presented as means ± s.d. (n=3). (**d-f**) BMDCs were pretreated with Y27632 or vehicle for 1 hour and then co-cultured with CT26.CL25 cells for 4 hours. After removal of un-engulfed tumour cells, the cells were incubated for additional 20 hours, and the percentage of $CD40^+$

or CD86$^+$ BMDCs was analysed by flow cytometry. (**d**) Representative flow cytometry results are shown. The gray peaks represent the isotype control. (**e**) The percentage and (**f**) relative MFI of CD40$^+$ or CD86$^+$ BMDCs are presented as means ± s.d. (n=3–4). Significance was determined by unpaired Student's *t*-test: ns, not significant.

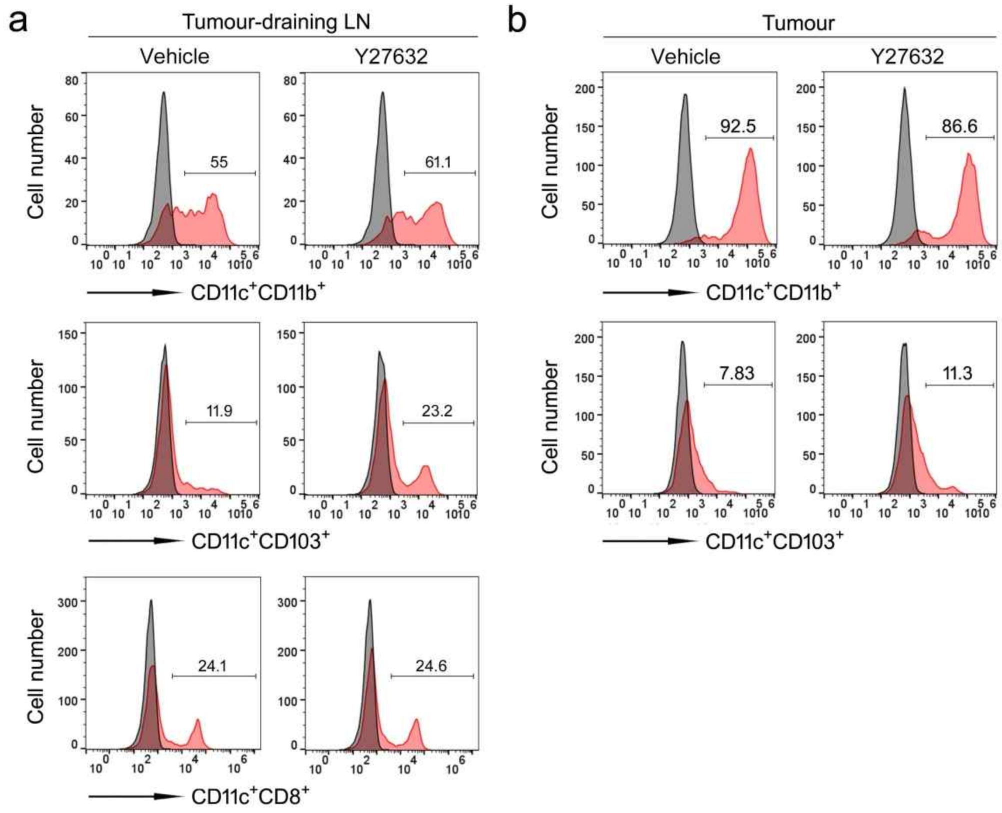

Supplementary Figure 12. DC subsets in B16F10-Ova tumour-bearing mice. B16F10-Ova tumour-bearing mice were injected (i.v.) with Y27632 (10 mg kg^{-1}) or vehicle (PBS). Twenty days after tumour inoculation, $CD11c^{+}$ DCs were isolated from tumour-draining LN (**a**) and tumours (**b**) though CD11c MACS sorting system. The percentages of $CD11c^{+}CD11b^{+}$, $CD11c^{+}CD103^{+}$, and $CD11c^{+}CD8^{+}$ DCs were analysed by flow cytometry using allophycocyanin-conjugated anti-CD11b (M1/70), anti-CD103 (2E7), and anti-CD8 (53-6.7) antibodies. Representative flow cytometry results are shown. The gray peaks represent the isotype control.

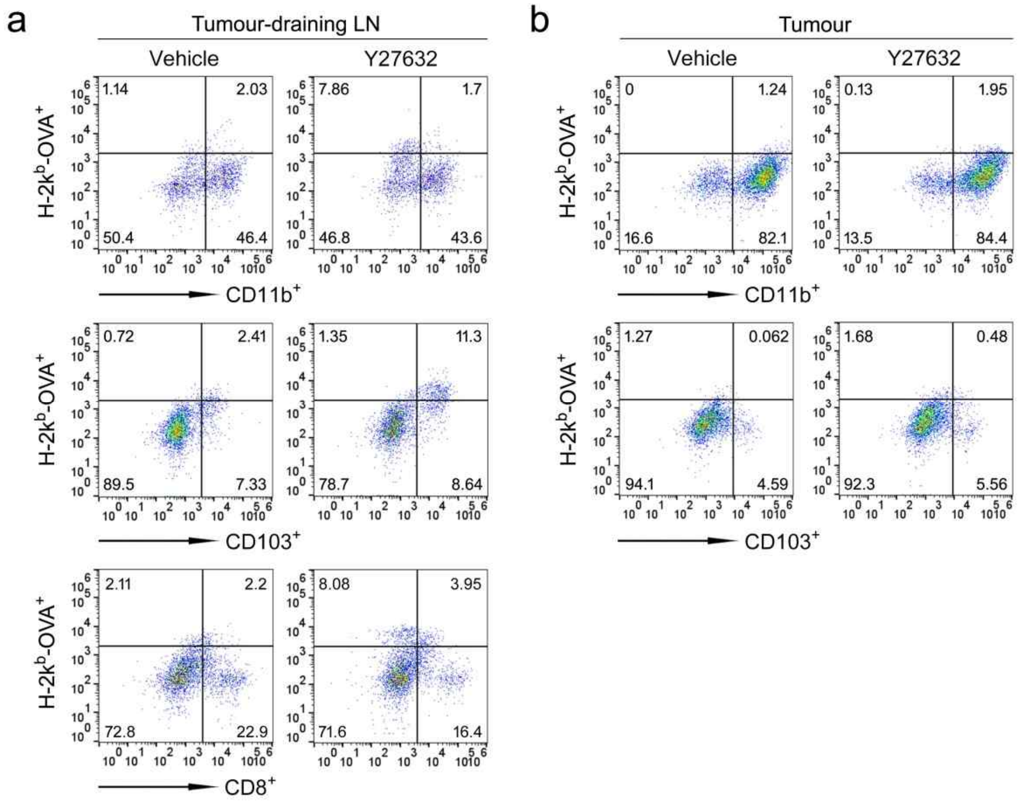

Supplementary Figure 13. Antigen presentation in DC subsets. B16F10-Ova tumour-bearing mice were injected (i.v.) with Y27632 (10 mg kg^{-1}) or vehicle (PBS). Twenty days after tumour inoculation, CD11c^{+} DCs were isolated from tumour-draining LNs (**a**) and tumours (**b**) though CD11c MACS sorting system. The percentage of H-2k^{b}-OVA^{+} cells in the indicated DC population was analysed by flow cytometry using a PE-conjugated anti-H-2k^{b}-OVA antibody and allophycocyanin-conjugated anti-CD11b (M1/70), anti-CD103 (2E7), or anti-CD8 (53-6.7) antibody. Representative flow cytometry results are shown.

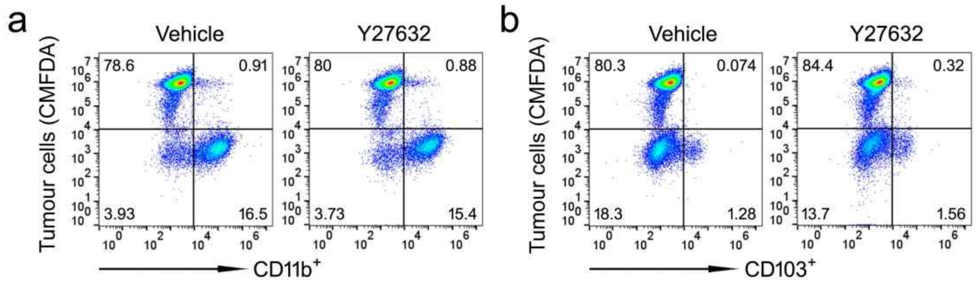

Supplementary Figure 14. Cancer cell phagocytosis in DC subsets. B16F10-Ova tumour-bearing mice were injected (i.v.) with Y27632 (10 mg kg^{-1}) or vehicle (PBS). Twenty days after tumour inoculation, $CD11c^{+}$ DCs were isolated from tumours though CD11c MACS sorting system. Then, $CD11c^{+}$ DCs were incubated with CMFDA-stained B16F10-Ova cells for 2 hours, and phagocytosis (%) in $CD11b^{+}$ and $CD103^{+}$ DCs were analysed by flow cytometry using allophycocyanin-conjugated anti-CD11b (M1/70) or anti-CD103 (2E7) antibody. Upper right quadrants indicate DCs harboring cancer cells. Representative flow cytometry plots are shown.

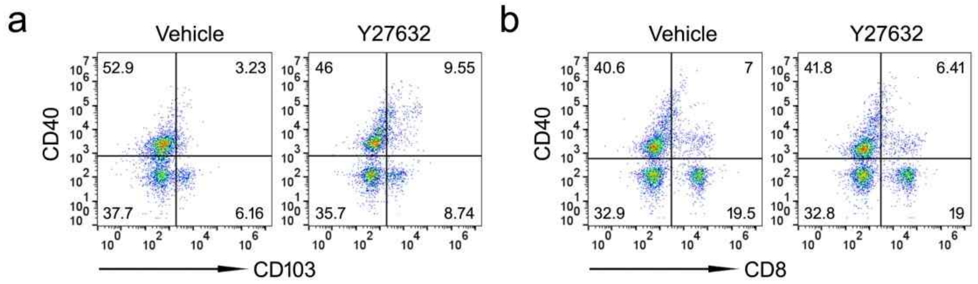

Supplementary Figure 15. DC maturation in CD103^{+} DCs. B16F10-Ova tumour-bearing mice were injected (i.v.) with Y27632 (10 mg kg^{-1}) or vehicle (PBS). Twenty days after tumour inoculation, CD11c^{+} DCs were isolated from tumour-draining LNs though CD11c MACS sorting system. The percentage of CD40^{+} cells in CD103^{+} or CD8^{+} DCs was determined by flow cytometry using PE-conjugated anti-CD40 (3-23) and allophycocyanin-conjugated anti-CD103 (2E7) or anti-CD8 (53-6.7) antibodies. Representative flow cytometry results are shown.

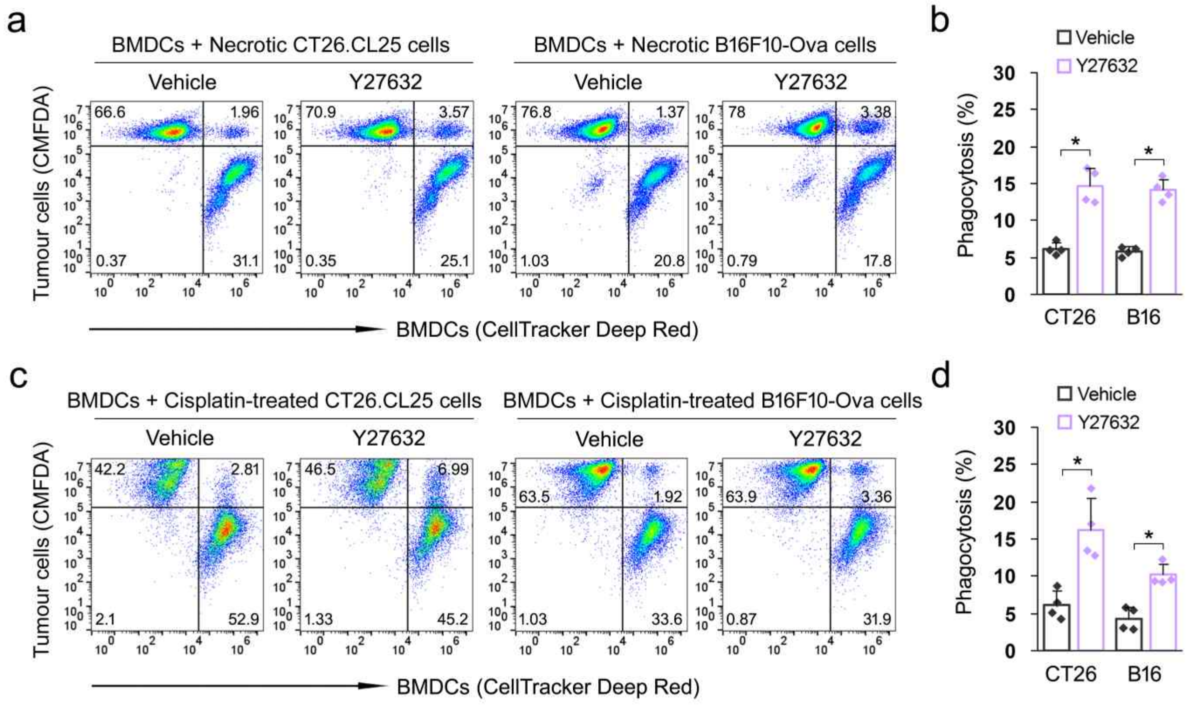

Supplementary Figure 16. ROCK blockade enhances engulfment of cells dying a non-immunogenic or necrotic cell death. (**a**,**b**) CellTracker Deep Red-labeled BMDCs were co-cultured with CMFDA-stained, necrotic CT26.CL25 or B16F10-Ova cells for 5 minutes, and then analysed by flow cytometry. (**a**) Representative flow cytometry plots. Upper right quadrant indicates BMDCs phagocytosing cancer cells. (**b**) The percentage of phagocytosis. Data are presented as means ± s.d. (n=4). (**c**,**d**) CellTracker Deep Red-labeled BMDCs were co-cultured with CMFDA-stained, cisplatin-treated CT26.CL25 or B16F10-Ova cells for 5 minutes, and then analysed by flow cytometry. (**c**) Representative flow cytometry plots. Upper right quadrant indicates BMDCs phagocytosing cancer cells. (**d**) The percentage of phagocytosis. Data are presented as means ± s.d. (n=4). **P* < 0.05; significance was determined by significance determined by Mann-Whitney test.

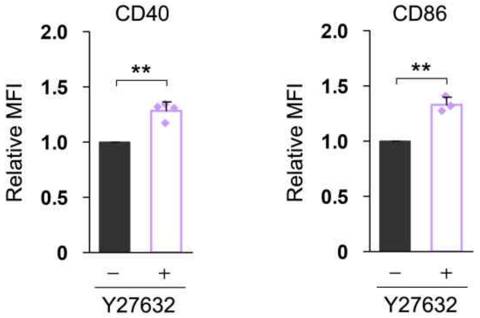

Supplementary Figure 17. DC maturation in BMDCs cocultured with Dox-treated cells. BMDCs were co-cultured with Dox-treated CT26.CL25 cells for 4 hours, and un-engulfed cells were then removed. After further incubation for 20 hours, the expression of CD40 or CD86 in BMDCs was analysed by flow cytometry. Relative CD40 or CD86 expression was calculated as the MFI. Data are presented as means ± s.d. (n=3–4). ***P* < 0.01; significance was determined by unpaired Student's *t*-test.

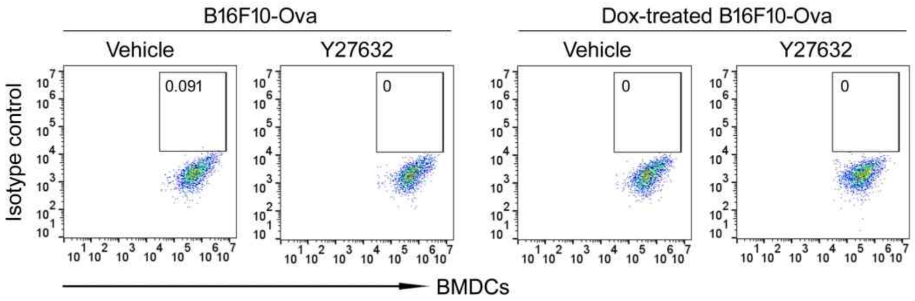

Supplementary Figure 18. Control experiment for antigen presentation in BMDCs. BMDCs were pretreated with Y27632 or vehicle and co-cultured with B16F10-Ova cells or Dox-treated B16F10-Ova cells for 4 hours and then un-engulfed cells were removed. After further incubation for 20 hours, the cells were stained with isotype control (PE-conjugated mouse IgG, κ isotype) for anti-H-2k^{b}-OVA antibody and analysed by flow cytometry. Representative flow cytometry plots are shown.

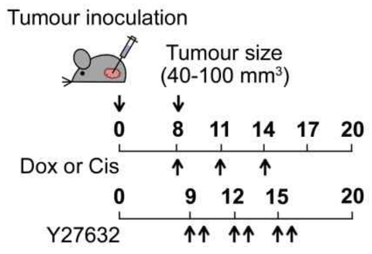

Supplementary Figure 19. Experimental schedule for combined therapy. Schematic diagram of experiments for investigating the combined effect of Y27632 and Dox or cisplatin (Cis) on tumour growth in B16F10-Ova tumour-bearing mice.

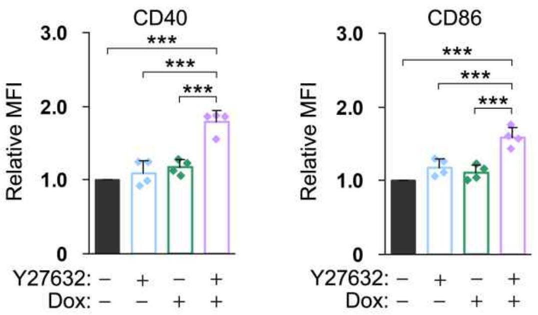

Supplementary Figure 20. DC maturation in DCs from tumour-bearing mice treated Y27632 and/or Dox. The relative MFI of CD40$^+$ and CD86$^+$ DCs among tumour-draining LN cells from B16F10-Ova tumour-bearing C57BL/6 mice treated Y27632 and/or Dox was analysed by flow cytometry. Data are presented as means ± s.d. (n=4). ***P < 0.001; significance determined by one-way ANOVA with Tukey's post-hoc test.

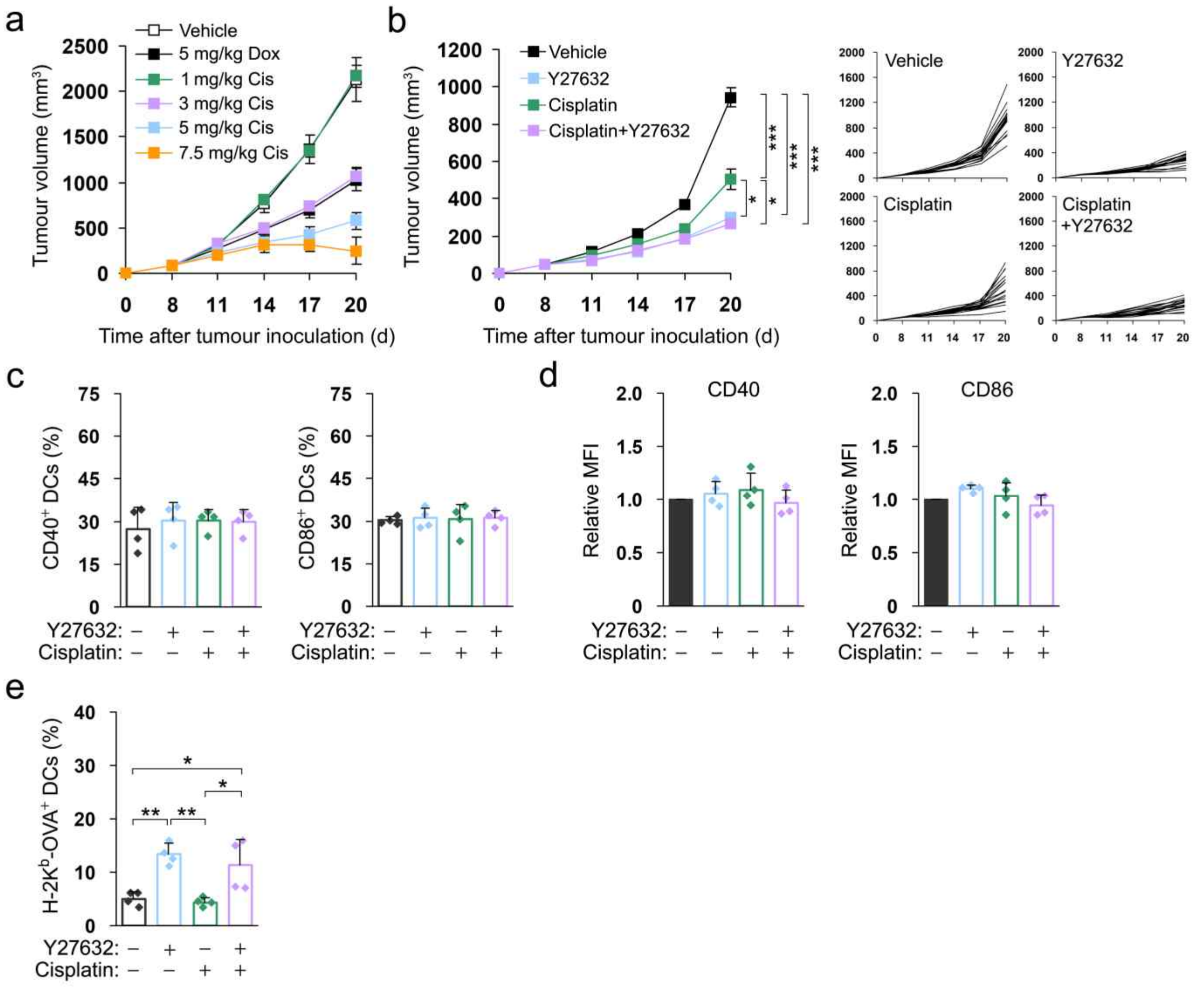

Supplementary Figure 21. Antitumour effect of combination of Y27632 and a non-ICD inducer. (**a**) B16F10-Ova tumour-bearing nude mice were injected (i.v.) with vehicle, Dox (5 mg kg^{-1}) or the indicated amount of cisplatin (Cis). Tumour volume was measured at the indicated times. Therapeutic effect of 5 mg kg^{-1} Dox was comparable to that of 3 mg kg^{-1} cisplatin. Data are presented as means ± s.e.m. (n=7) (**b-e**) B16F10-Ova tumour-bearing C57BL/6 mice were injected (i.v.) with Y27632 (10 mg kg^{-1}) and/or cisplatin (3 mg kg^{-1}), as indicated in Supplementary Fig. 19. (**b**) Tumour volume was measured at the indicated times. Data are presented as means ± s.e.m. (n=18) (left panel) and tumour growth curves of individual mice (right panels). (**c**,**d**) The percentage (**c**) and relative MFI (**d**) of CD40$^+$ and CD86$^+$ DCs among tumour-draining LN cells from B16F10-Ova tumour-bearing C57BL/6 mice treated Y27632 and/or cisplatin were analysed by flow cytometry. Data are presented as means ± s.d. (n=4). (**e**) Tumour-draining LN cells were isolated from B16F10-Ova tumour-bearing C57BL/6 mice treated with Y27632 and/or cisplatin, and the percentage of H-2k^b-OVA$^+$ DCs was analysed by flow cytometry using an antibody recognizing a complex of the

ovalbumin peptide (SIINFEKL) with H-2k^b (MHC I). Data are presented as means ± s.d. (n=4). *P < 0.05, **P < 0.01, ***P < 0.001; significance determined by Kruskal-Wallis test with Bonferroni correction (**b**), or one-way ANOVA with Tukey's post-hoc test (**c**-**e**).
